# TLR4/NF-κB signaling-mediated neuroinflammation is associated with gut microbiota dysbiosis in a mouse model of Parkinson’s disease

**DOI:** 10.3389/fimmu.2026.1672241

**Published:** 2026-01-30

**Authors:** Ruqi Zhang, Minghan Tian, Yangyang Wu, Chen Yang, Xiaoyu Shi, Shengchun Wang

**Affiliations:** 1 College of Acupuncture, Moxibustion and Tuina, Shandong University of Traditional Chinese Medicine, Jinan, China; 2 Department of Rehabilitation Medicine, Linyi People’s Hospital Affiliated to Shandong Second Medical University, Linyi, China; 3 Department of Acupuncture, Physiotherapy and Rehabilitation, Shandong Provincial Hospital Affiliated to Shandong First Medical University, Jinan, China

**Keywords:** gut microbiota, inflammatory response, microbiota-gut-brain axis, Parkinson’s disease, TLR4/NF-κB signaling pathway

## Abstract

**Introduction:**

Dysbiosis of the microbiota-gut-brain axis contribute to the neurodegenerative process of Parkinson’s disease (PD), and dysbiosis and inflammatory responses represent key mechanisms. This study aims to explore the structural changes in the composition of the gut microbiota and the alterations in the inflammatory response mediated by the TLR4/NF-κB pathway in a rotenone-induced PD mouse model, as well as the correlation between the two.

**Methods:**

The motor coordination and spontaneous locomotor activity of the PD mouse model were evaluated using the Rota-Rod test, pole climbing test and open field test. The expression of α-synuclein (α-syn) and the activation status of the TLR4/NF-κB pathway were analyzed by western blot, quantitative real-time polymerase chain reaction (RT-qPCR) combined with immunohistochemistry. Enzyme-linked immunosorbent assay (ELISA) was used to quantitatively detect the levels of LPS and pro-inflammatory indicators TNF-α, IL-1β and IL-6. The diversity, composition structure and differential abundance of the gut microbiota were analyzed by 16S rRNA sequencing, and correlation analysis was conducted between some microbiota and inflammatory indicators related to the activation of the TLR4/NF-κB signaling pathway.

**Results:**

Mechanistic investigation revealed that rotenone activated the TLR4/NF-κB signaling pathway in the midbrain substantia nigra (SN) and colon tissues, accompanied by a significant increase in LPS levels and pro-inflammatory indicators. 16S rRNA sequencing analysis revealed that the alpha diversity of the gut microbiota were reduced in the model group, the beta diversity structure was altered. In terms of microbiota composition, at the phylum level, the relative abundance of Bacteroidota decreased, while Actinobacteria and Tenericutes increased. At the family level, the relative abundance of *Lachnospiraceae* and *Bacteroidaceae* decreased, while the relative abundance of *Erysipelotrichaceae* and *Akkermansiaceae* increased. Correlation analysis indicated that the relative abundance of specific bacterial families was significantly correlated with PD motor function indicators, the expression levels of α-syn mRNA in the midbrain SN, the TLR4/NF-κB pathway, and inflammatory indicators.

**Conclusion:**

This study demonstrates a key role of the TLR4/NF-κB signaling pathway in the microbiota-gut-brain axis of a rotenone-induced PD mouse model, where gut microbiota dysbiosis exhibits a significant correlation with inflammation induced by TLR4/NF-κB activation.

## 1 Introduction

Parkinson’s disease (PD) is a common chronic progressive neurodegenerative disorder. Its clinical manifestations involve complex symptom clusters, mainly characterized by typical motor symptoms such as bradykinesia, resting tremor, muscle rigidity and postural instability and gait disorders (1, 2). At the same time, it presents with non-motor symptoms such as neuropsychiatric symptoms, cognitive dysfunction, sleep disorders, affective disorders and autonomic nerve dysfunction, which also seriously affect the quality of life of patients (3, 4). Its core pathological features are manifested as the progressive degeneration and loss of dopaminergic neurons in the striatum pathway of midbrain substantia nigra (SN), as well as the formation of lewy bodies by abnormal aggregation of α-synuclein (α-syn) (5, 6).

Increasing evidence implicates the microbiota-gut-brain axis in PD pathogenesis. Alterations in gut microbiota composition are frequently observed in PD patients (7). This gut dysbiosis is proposed to contribute to PD through multiple mechanisms, including the promotion of systemic and central inflammation. Specifically, microbial products, such as lipopolysaccharide (LPS), may activate pro-inflammatory signaling pathways. The toll-like receptor 4 (TLR4)/nuclear factor-κB (NF-κB) signaling pathway is a critical mediator of innate inflammatory responses and neuroinflammation. Activation of this pathway in microglia, the resident immune cells of the brain, can drive the production of pro-inflammatory indicators, which may contribute to dopaminergic neuron degeneration (8, 9). Recent studies have begun to link gut microbiota dysbiosis to the regulation of neuroinflammation via the TLR4/NF-κB pathway in the context of PD (10). However, the precise mechanistic relationship between specific gut microbiota alterations and TLR4/NF-κB-mediated neuroinflammation remains incompletely defined.

Therefore, this study aims to characterize PD-associated microbiota signatures critical in pathogenesis, identify microbiome biomarkers correlating with neurological and intestinal inflammatory indicators, and also provide foundations for the development of precision microbiota-targeted therapies.

## 2 Materials and methods

### 2.1 Establishment of rotenone induced PD mouse model

Twelve 8-week-old (weighing 18-20 g) male C57BL/6J mice (purchased from Beijing Vital River Laboratory Animal Technology Co., Ltd.) were separately caged under specific pathogen-free (SPF) conditions at the Animal Experimental Center of Shandong Provincial Hospital Affiliated to Shandong First Medical University. The license number for use is SYXK (Lu) 2023-0012. Animals were maintained at 24 ± 2°C with 50 ± 10% relative humidity under 12-h light/dark cycles, with ad libitum access to food and water. After a 7-day acclimation period under these conditions, experiments commenced. This experimental procedure has been approved by the Animal Ethics Committee of Shandong Provincial Hospital Affiliated to Shandong First Medical University (NO. NSFC2024-042).

**Figure 1:**
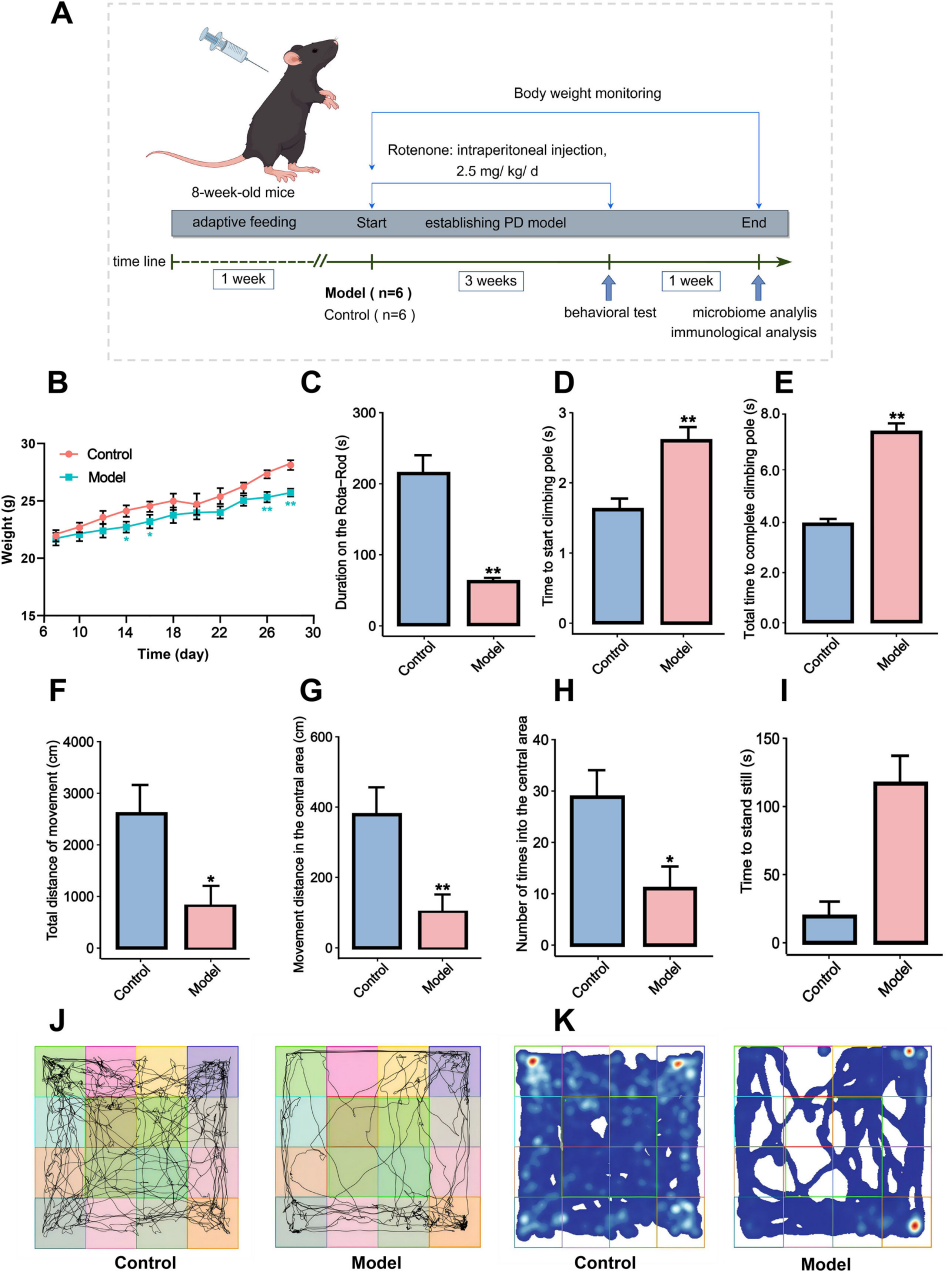
General state, motor symptoms and behavioral changes of rotenone-induced PD mouse model. **(A)** Animal experiment operation procedures. **(B)** Changes in mouse body weight. **(C)** Rota-Rod test. **(D, E)** Pole climbing test. **(F-I)** Open field test. **(J)** Movement trajectory map of the open field experiment. **(K)** Heatmap of the movement trajectory in the open field experiment. All data are presented as mean ± SD (n = 6). Compared with the control group, *P < 0.05, **P < 0.01.

Twelve mice were randomly divided into the control group and the model group (n=6/group). PD mouse models were established by chronic low-dose subcutaneous injection of rotenone. Rotenone powder (Sigma-Aldrich, USA), dissolved in dimethyl sulfoxide (Gibco, USA) at 0.05 mg/mL, was administered via subcutaneous injection in the dorsocervical region at a daily dose of 2.5 mg/kg for 3 consecutive weeks. The control group received equivalent-volume distilled water via oral gavage. The specific operation process of the animal experiment is shown in **Figure 1A**. After the modeling was completed, successful induction was confirmed through behavioral assessments. Body weights were recorded every 48 h until behavioral testing completion. All mice underwent 12-h fasting after the final behavioral test. Mice were anesthetized via intraperitoneal injection of 1% pentobarbital sodium (75 μL/g body weight) and transcardially perfused with ice-cold 0.9% saline until effluent ran clear and limbs stiffened. Brains, intestines and feces were collected and stored respectively in a −80°C refrigerator for subsequent analysis (10, 11).

### 2.2 Behavioral experiment

#### 2.2.1 Balance coordination function detection

Each mouse underwent a Rota-Rod test followed by a pole climbing test. For the Rota-Rod test, the mouse was placed on a rotating rod that accelerated from 4 r/min to 40 r/min over a 2-minute period and then maintained at 40 r/min for 5 minutes. The latency to fall was automatically recorded by the instrument (12, 13). In the climbing pole test, a rough-surfaced metal pole (50 cm in length, 2 cm in diameter) with a small ball affixed to the top served as the starting point. At the beginning of the test, each mouse was held by the tail and placed head-down on the top of the pole (12). The time taken for the mouse to turn around and descend to the base of the pole was recorded. The mice received training before tests. Each mouse underwent three trials for both tests, with a 30-minute interval between trials. The average of the three trials was used for analysis.

#### 2.2.2 Autonomous motor function detection

An open field test was performed using a standard open field apparatus (40 × 40 × 40 cm). The floor of the apparatus was evenly divided into 16 equal squares. Each mouse was gently placed in the central zone (the four central squares) and allowed to explore freely for 5 minutes. The camera for recording was installed above the device. The following parameters were recorded by a motion tracking system: movement trajectory, total distance traveled, distance traveled within the central area, number of entries into the central area, and duration of immobility (12, 14). After each trial, the apparatus was cleaned with 75% ethanol to eliminate residual odors and prevent interference with subsequent behavioral assessments.

### 2.3 Sample collection and index detection

#### 2.3.1 Western blot

After euthanasia by decapitation under deep anesthesia, the entire brain tissue was rapidly isolated and placed on an ice table. Referring to the mouse brain atlas, midbrain tissue containing SN was dissected from the coronal plane of the frozen sector. Approximately one-third of the tissue was collected. Additionally, a 2 cm segment of colon tissue located 7 cm proximal to the anus was excised. Samples were kept on ice for 30 minutes to allow lysis. The tissues were homogenized by ultrasonication, followed by centrifugation at 12,000 r/min for 15 minutes at 4°C. The resulting supernatant was collected for protein analysis.

Protein concentration was determined using the bicinchoninic acid (BCA) assay kit (P0012, Beyotime Biotechnology, China) as described previously (15). Proteins were separated by sodium dodecyl sulfate polyacrylamide gel electrophoresis (SDS-PAGE) using a gel preparation kit (G2003, Servicebio, China), and then transferred onto a PVDF membrane (IPVH00010, Millipore, China). The membrane was blocked with 5% skim milk (G5002, Servicebio, China) at room temperature for 1.5 hours, followed by incubation with primary antibodies overnight at 4°C. The following primary antibodies were used: rabbit monoclonal anti-α-synuclein (1:1000, A20407, ABclonal, China), rabbit polyclonal anti-TLR4 (1:2000, A5258, ABclonal, China), rabbit polyclonal anti-NF-κB1 (1:1000, A6667, ABclonal, China), and mouse monoclonal anti-GAPDH (1:10,000, 60004-1-Ig4, Proteintech Group, China). After washing, membranes were incubated with HRP-conjugated polyclonal secondary antibodies: goat anti-rabbit (1:10,000, ZB-2301, ZSGB, China) and goat anti-mouse (1:10,000, ZB-2305, ZSGB, China) for 1 hour at room temperature. Protein bands were visualized using an automated chemiluminescence imaging system (Tanon5200, China), and the optical density of each band was analyzed semi-quantitatively using AlphaEaseFC software (Alpha Innotech, USA).

#### 2.3.2 Quantitative real-time polymerase chain reaction

Approximately one-third of the SN tissue from the midbrain and a 2 cm segment of colon tissue located 7 cm proximal to the anus were excised. Total RNA was extracted using Trizol Reagent (CW0580S, Cowin Biotechnology, China) in accordance with the manufacturer’s protocol and relevant literature (16). The mRNA was further purified using the mRNA Ultra-Pure Extraction Kit (CW0581M, Cowin Biotechnology, China), and its concentration and purity were assessed using a spectrophotometer. Complementary DNA (cDNA) was synthesized by reverse transcription using the SYBR^®^ Green Premix Pro Taq HS qPCR Kit (AG11701, Aikori Biotechnology, China), following the manufacturer’s instructions. Quantitative real-time polymerase chain reaction (RT-qPCR) was then performed using the ABI 7500 Real-Time PCR System (Applied Biosystems, USA). GAPDH was used as the internal reference gene, and relative expression levels of target genes were calculated using the 2^–ΔΔ^Ct method (17). The primer sequences are listed in **Table 1**.

**Table 1:** Paired primers for RT-qPCR.

Gene	Primer sequence (5′-3′)	
	Forward	Reverse
α-syn	AGTGGTGACTGGTGTGACA	TCTTGGTAGCCTTCCTCTGAA
TLR4	GTGCCAGTCAGGGTCATTCA	ACTCCCCAGCCCTTTATGGA
NF-κB	CCTCTGGCGAATGGCTTTAC	TGCTTCGGCTGTTCGATGAT
GAPDH	GGTGAAGGTCGGTGTGAACG	CTCGCTCCTGGAAGATGGTG

#### 2.3.3 Enzyme-linked immunosorbent assay

The remaining midbrain tissue and a 2 cm segment of colon tissue located 7 cm proximal to the anus were used for analysis. According to the manufacturer’s instructions for the mouse tumor necrosis factor-α (TNF-α) (F2132-B), interleukin (IL)-1β (F2040-B), IL-6 (F2163-B), and LPS (F2631-B) ELISA kits (Shanghai Fanke Industrial Co., Ltd., China), standards were diluted to a concentration of 10 μg/mL. Absorbance was measured using a multifunctional microplate reader (multiskan sky, Thermo Fisher, USA), and dual-wavelength correction was applied. After baseline correction using the blank wells, the optical density (OD) values of each sample well were recorded (18).

#### 2.3.4 Immunohistochemistry

A 2 cm segment of colon tissue located 7 cm proximal to the anus was excised and fixed in 4% paraformaldehyde for paraffin embedding. The paraffin blocks were sectioned into 3 μm slices. Sections were baked in an oven at 65°C for 2 hours for dewaxing, followed by ethanol dewaxing and graded dehydration. Add the primary antibody (α-syn, 1: 100, 10842-1-AP, Proteintech Group, China) and incubate overnight at 4 °C. Wash again three times with PBS for 5 minutes each time, add the secondary antibody (1:200), and incubate at room temperature for 40 minutes. Finally, they were subjected to gradient ethanol dehydration, xylene transparency and neutral gum sealing treatment. Photos were taken under an optical microscope (BX53M, Olympus, Japan) for observation. The cumulative absorbance value was determined using ImageJ software (11).

#### 2.3.5 16S rRNA sequencing and bioinformatics analysis

Under aseptic conditions, approximately 1 g of fecal sample was collected from each mouse’s colon and placed into a sterile EP tube. The genomic DNA of the samples was extracted by the CTAB method. The V3-V4 region of the 16S rRNA gene was amplified by 341F/806R primers. After library preparation with the TruSeq^®^ DNA PCR-Free Sample Preparation Kit (Illumina, USA), libraries were quantified via Qubit (V2.0) and sequenced on the Illumina NovaSeq 6000 platform (19, 20). Sequencing data were uploaded to the Majorbio Cloud Platform (https://www.majorbio.com/tools) for bioinformatic analysis. This platform employed Trimmomatic (v0.36) for quality control of raw sequencing reads, followed by FLASH (v1.2.11) for sequence assembly. The assembled sequences were subsequently analyzed using QIIME (v1.9.1). Sequences were clustered into operational taxonomic units (OTUs) at 97% similarity using UPARSE (v11) and USEARCH (v11), followed by chimera removal. Aligned against the SILVA database (SSU128), sequences were taxonomically classified using the RDP classifier with a Bayesian algorithm at a 70% confidence threshold (21). Alpha diversity metrics were calculated using Mothur (v1.30.2), while beta diversity, species composition and differential analysis, univariate network analysis, and Spearman correlation were performed using R (v3.5.1).

### 2.4 Statistical methods

Statistical analyses were performed using GraphPad Prism 8.0. Data visualization was conducted using the Majorbio Cloud Platform (http://cloud.majorbio.com), ImageGP (https://www.bic.ac.cn/BIC/#/), and the Bioinformatics online platform (https://www.bioinformatics.com.cn/). Student’s t-test, Wilcox’s rank sum test and Spearman correlation were respectively used for analysis. A *P* value <0.05 was considered statistically significant.

## 3 Results

### 3.1 General state deterioration and motor dysfunction of rotenone induced PD mouse model

To investigate the pathogenic mechanism and correlation between the inflammatory response and gut microbiota in PD mice induced by rotenone, a PD mouse model was established (**Figure 1A**). After the PD model was established, the body weight of mice in the PD model group was lower than that of the control group (**Figure 1B**). Morphologically, the model mice exhibited visibly unkempt, yellow, and dirty fur, sialorrhea, hunchback posture, weakened grasping reflexes, reduced activity, slow movement, unsteady gait, lateral circling, and in some cases, hind limb paralysis. In the fourth week following modeling, three behavioral tests—Rota-rod test, pole test, and open field test—were performed to assess motor function and confirm successful model establishment. Compared with the control group, model mice spent significantly less time on the Rota-Rod test (*P* < 0.01, **Figure 1C**), and exhibited prolonged times to state and complete pole climbing test (*P* < 0.01, **Figures 1D, E**). In the open field test, the total movement distance, movement distance in the central area, and the number of entries into the central area were significantly reduced in the model group (*P* < 0.05 or *P* < 0.01, **Figures 1F–H**), while no significant difference was observed in time to stand still (*P* > 0.05, **Figure 1I**). In addition, movement trajectory maps and Heatmaps further confirmed that rotenone-induced mice exhibited marked deficits in spontaneous locomotion (**Figures 1J, K**).

### 3.2 Abnormal aggregation of α-syn in rotenone induced PD mouse model

**Figure 2:**
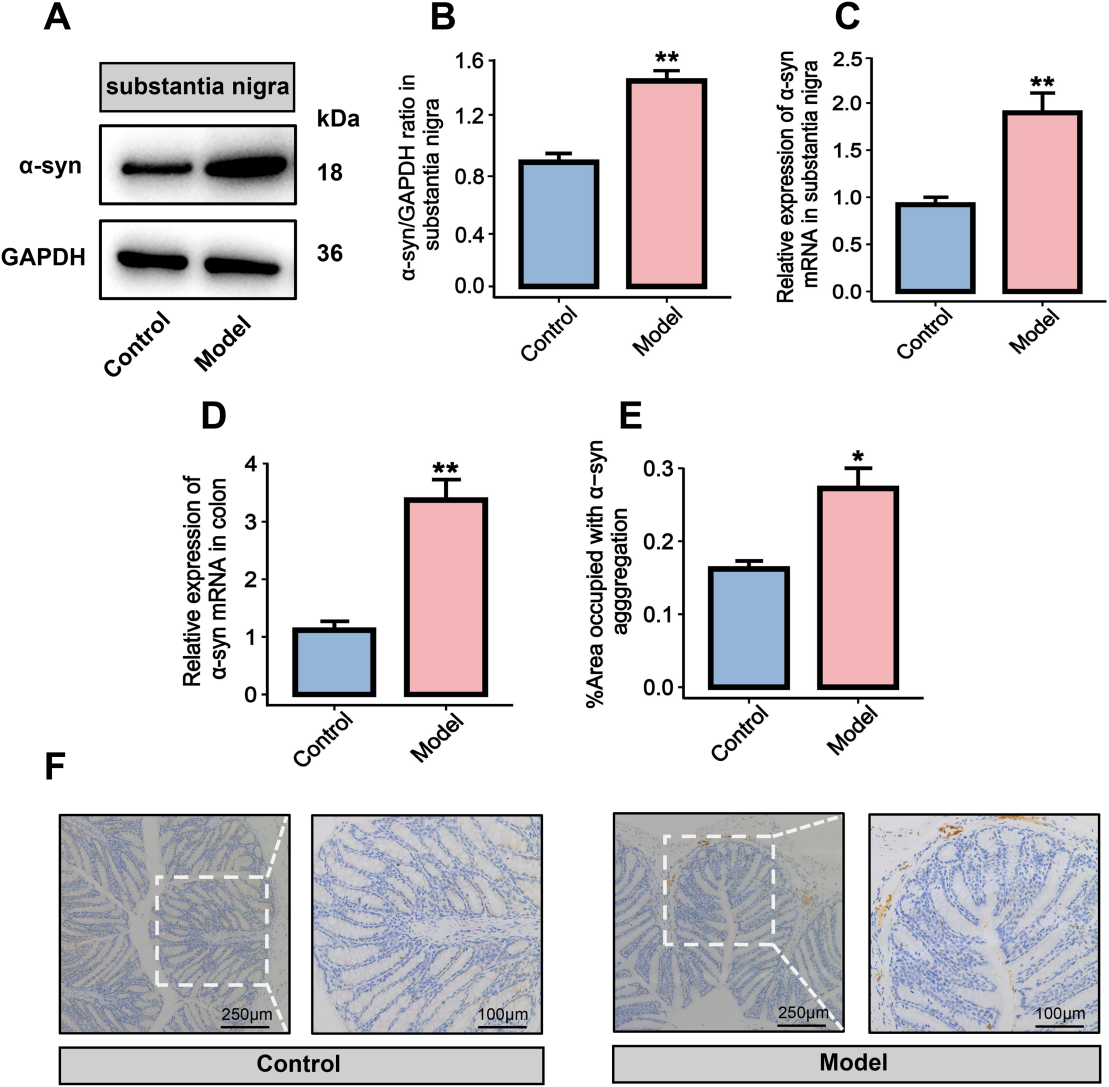
Histological characteristics of α-syn in rotenone-induced PD mouse model. **(A)** Western blot brands. **(B)** Analysis of the western blot optical density value. **(C, D)** RT-qPCR analysis. **(E)** %Area occupied with α-syn agggregation. **(F)** Immunohistochemistry of α-syn (brown imaging) in the colon. All data are presented as mean ± SD (n = 6). Compared with the control group, *P < 0.05, **P < 0.01.

To further verify this histological alteration of the PD mouse model, western blot was used to detect α-syn in the midbrain SN of the model group mice. The results showed that compared with the control group, the expression of α-syn protein in the model group mice was abnormally aggregated (*P* < 0.01, **Figures 2A, B**). RT-qPCR analysis also showed markedly increased α-syn mRNA levels in the SN of model mice (*P* < 0.01, **Figure 2C**). Immunohistochemical staining of colon tissues showed elevated α-syn expression in the model group compared with the control group (*P* < 0.05, **Figures 2E, F**). RT-qPCR analysis confirmed significantly upregulated α-syn mRNA expression in the colon of model mice (*P* < 0.01, **Figure 2D**).

### 3.3 TLR4/NF-κB signaling pathway was activated in SN and colon of rotenone induced PD mouse model

**Figure 3:**
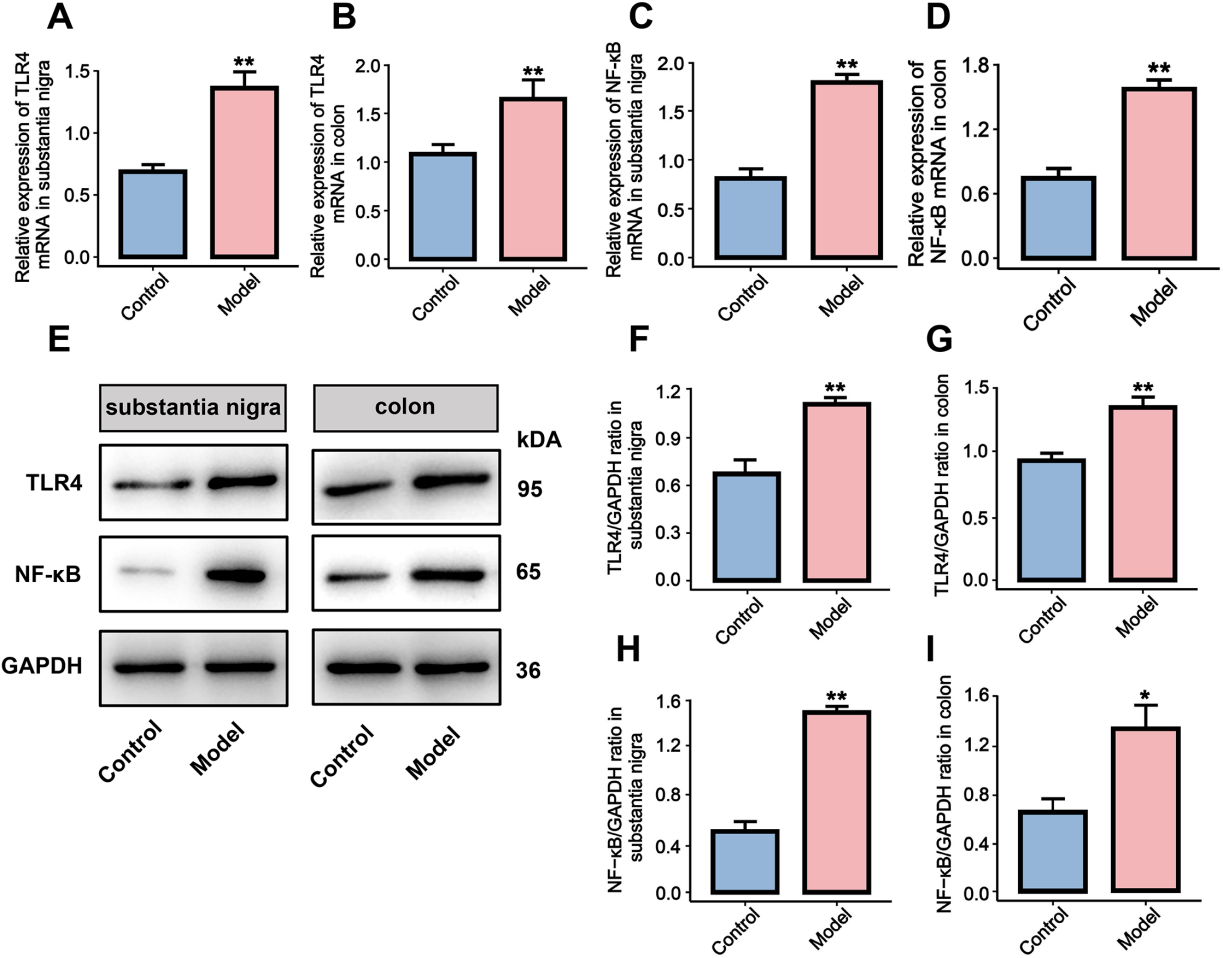
The effect of rotenone induced-PD mouse model on the TLR4/NF-κB signaling pathway in the midbrain SN and colon. **(A–D)** RT-qPCR analysis. **(E)** Western blot brands. **(F–I)** Analysis of western blot optical density values. All data are presented as mean ± SD (n = 6). Compared with the control group, *P < 0.05, **P < 0.01.

To assess the activation status of the TLR4/NF-κB pathway, we examined the expression levels of relevant genes and proteins in the SN and colon using RT-qPCR and western blot analyses. RT-qPCR results showed that compared with the control group, the expression levels of TLR4 mRNA and NF-κB mRNA were significantly elevated in the SN of the model group (*P* < 0.01**, Figures 3A, C**). Similarly, TLR4 mRNA and NF-κB mRNA expression levels were also markedly increased in the colon (*P* < 0.01, **Figures 3B, D**), consistent with the trends observed in the midbrain. Western blot analysis further confirmed that the protein expression levels of TLR4 and NF-κB were significantly higher in both the SN and colon tissues of the rotenone-induced mice compared to the control group (*P* < 0.01 or *P* < 0.05, **Figures 3E–I**).

### 3.4 Neuroinflammation and intestinal inflammation appeared in rotenone induced PD mouse model

**Figure 4:**
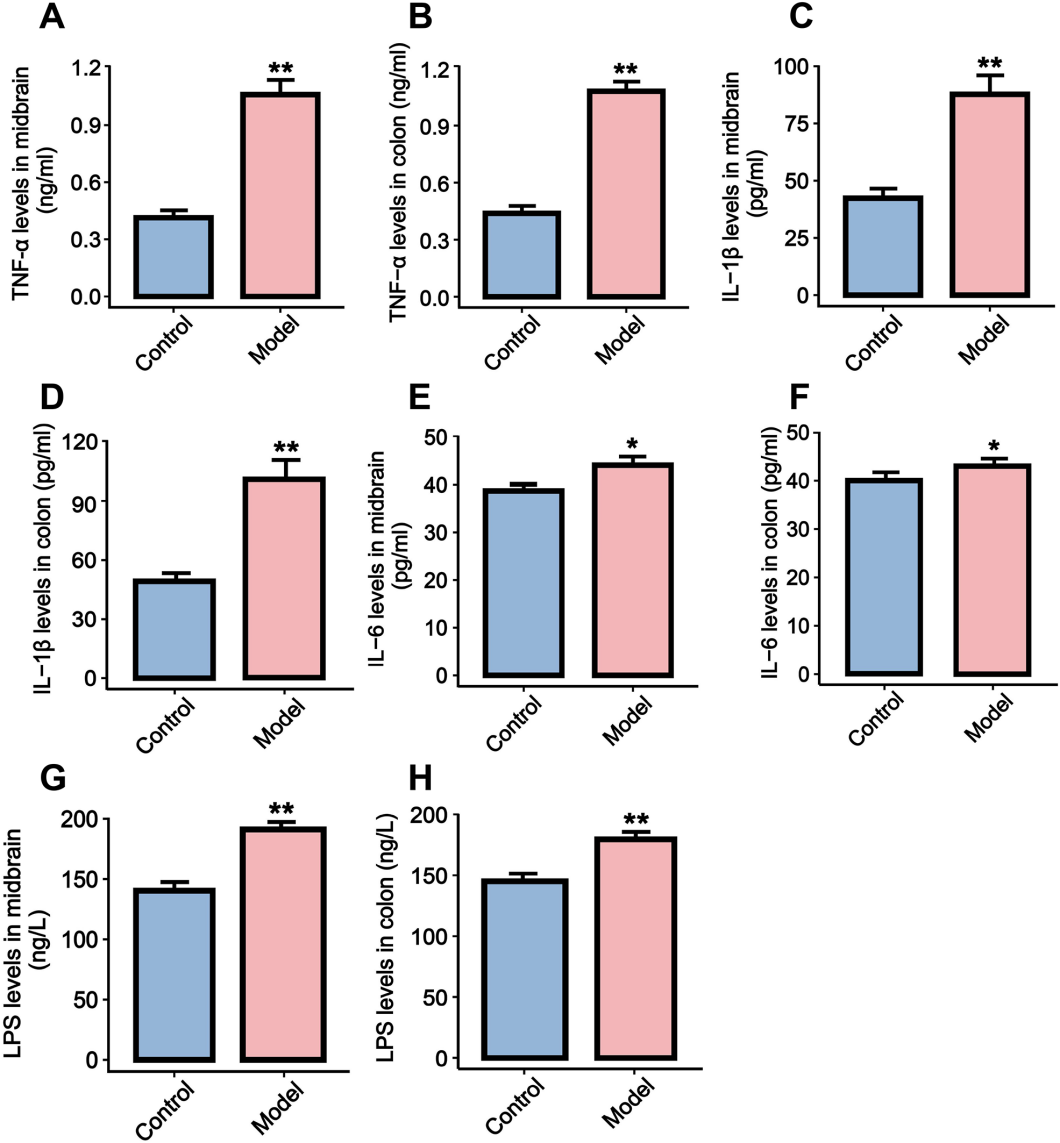
The effects of rotenone-induced PD mouse models on neuroinflammation and intestinal inflammation. The changes in the levels of TNF-α **(A, B)**, IL-1β **(C, D)**, IL-6 **(E, F)** and LPS **(G, H)** in midbrain and colon tissues were analyzed by ELISA. All data are presented as mean ± SD (n = 6). Compared with the control group, *P < 0.05, **P < 0.01.

Evidence suggested a bidirectional relationship between neuroinflammation in the central nervous system and inflammation in the gastrointestinal tract. On one hand, LPS derived from the gut may enter the circulation and activate the TLR4/NF-κB signaling pathway in the midbrain, leading to dopaminergic neuronal degeneration. Therefore, we measured LPS levels in the SN and colon using ELISA. The results showed that LPS content in both tissues was significantly increased in the model group compared to the control group (*P* < 0.01, **Figures 4G, H**). On the other hand, once α-syn entered the brain parenchyma, microglia pattern recognition receptors such as TLR4 could be activated, triggering the assembly of NLRP3 inflammasome and the release of pro-inflammatory indicators (TNF-α, IL-1β, IL-6), thereby forming a neuroinflammatory microenvironment. These inflammatory indicators may promote intestinal inflammation through retrograde vagus nerve signaling, creating a self-reinforcing pathological loop. Therefore, we measured the levels of TNF-α, IL-1β, and IL-6 in both the SN and colon tissues by ELISA. The results significantly elevated levels of all three cytokines in the model group compared to controls (*P* < 0.01 or *P* < 0.05, **Figures 4A–F**), consistent with the observed increase in LPS levels.

### 3.5 Species annotation and evaluation of gut microbiota in rotenone induced PD mouse model

**Figure 5:**
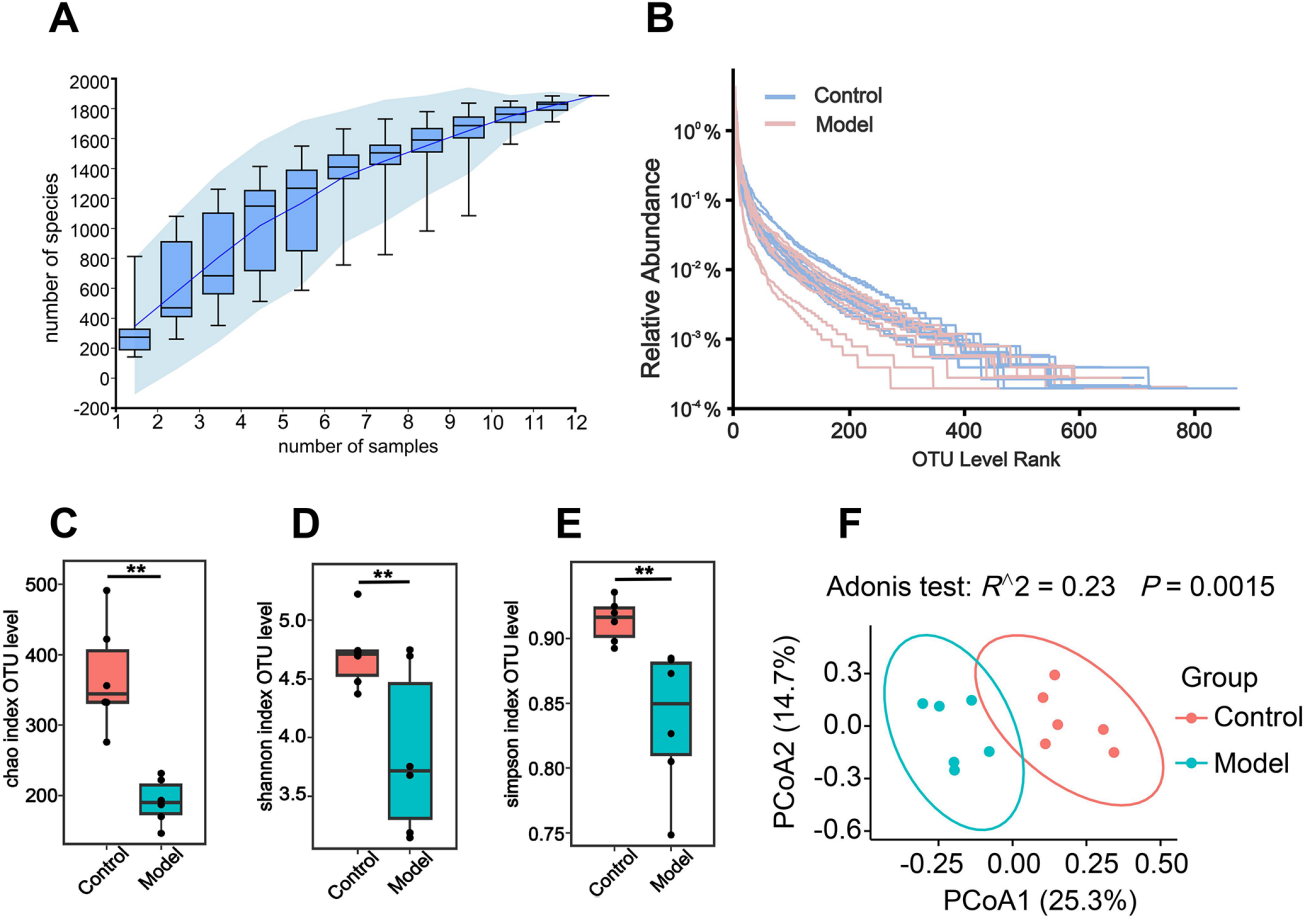
Species annotation and evaluation of gut microbiota in rotenone-induced PD mouse models. **(A)** Species accumulation curve. **(B)** The Rank-abundance curve. **(C)** Chao index. **(D)** Shannon index. **(E)** Simpson index. **(F)** PCoA analysis. The Chao, Shannon, and Simpson indices belong to alpha diversity analysis, while PCoA analysis belongs to beta analysis. Each boxplot represents the median, interquartile range, minimum and maximum values (n = 6). Compared with the control group, **P < 0.01.

To clarify how the gut microbiota affects PD and the correlation between the quantitative and structural changes of the microbiome community and inflammatory indicators in the rotenone induced PD mouse models, fecal samples from both control and model groups underwent 16S rRNA sequencing. The OTU species analysis after subsampling the minimum number of sample sequences showed that there were a total of 12 samples in 2 groups of sequencing data. The sequence length of the microbiota was distributed between 400 and 500 bp, which was basically consistent with the sequence length of the V3-V4 region of 16S rRNA. Species accumulation curves plateaued as sample size increased, indicating adequate sampling depth (**Figure 5A**). Rank−abundance curves demonstrated broad distribution and gentle decline, reflecting high species richness and evenness (**Figure 5B**). Alpha diversity indices (Chao1, Shannon, Simpson) were all significantly lower in the model group compared with the control group (*P* < 0.01, **Figures 5C–E**), implying reduced microbial richness and diversity in PD mice. Beta diversity analysis using principal coordinates analysis (PCoA) revealed clear separation between groups, and adonis testing confirmed significant inter-group differences in microbiota composition (*R* = 0.23, *P* = 0.0015, **Figure 5F**).

### 3.6 Gut microbiota dysbiosis in rotenone induced PD mouse model

**Figure 6:**
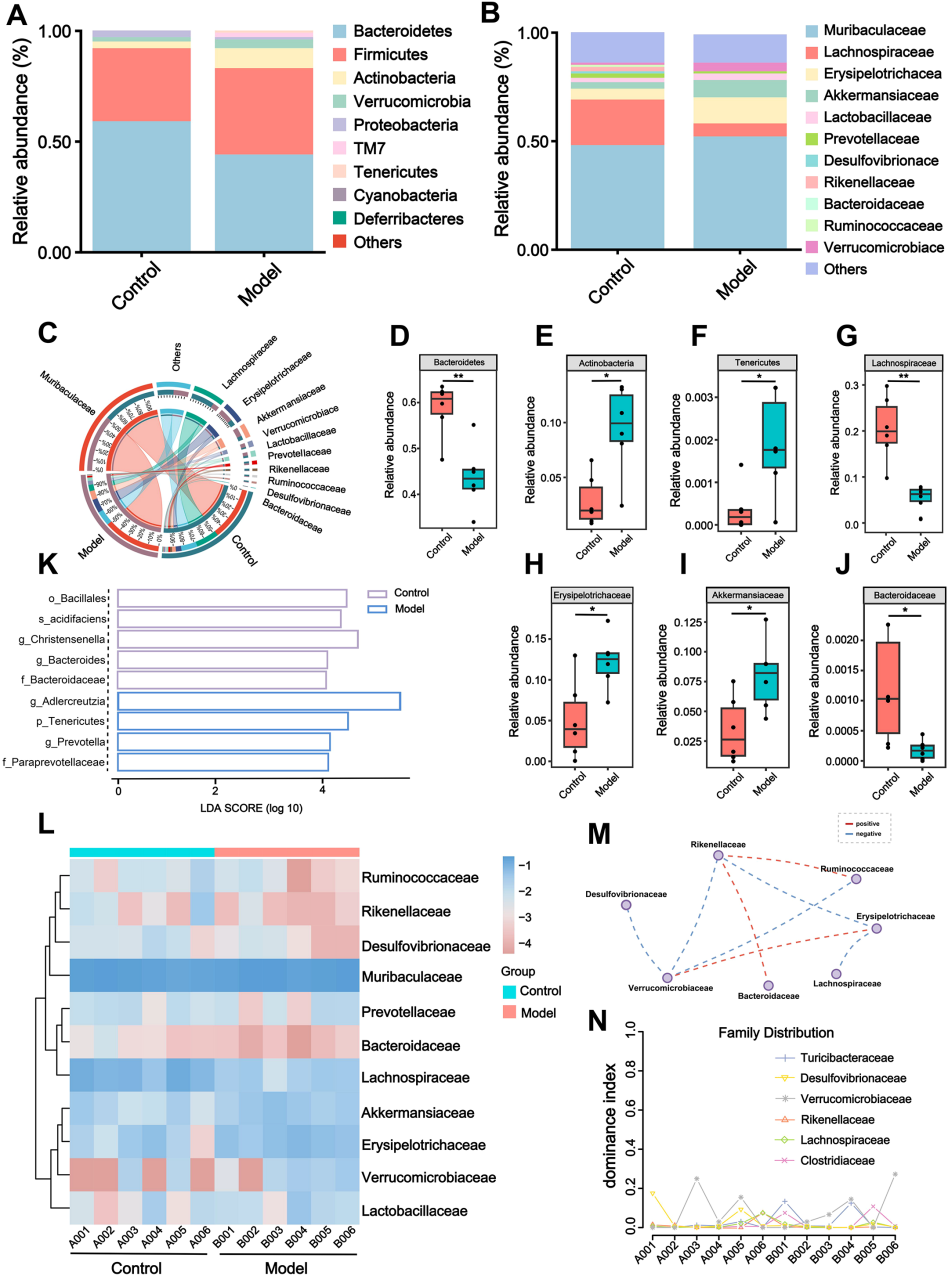
Gut microbiota dysbiosis in rotenone-induced PD mouse model. **(A, B)** Bar charts of species composition at the phylum and family levels. **(C)** Circos diagram of species composition at the family level. The relative abundance of different bacteria such as Bacteroidota **(D)**, Actinobacteria **(E)**, Tenericutes **(F)**, *Lachnospiraceae***(G)**, *Erysipelotrichaceae***(H)**, *Akkermansiaceae***(I)** and *Bacteroidaceae***(J)** in the model group and the control group was compared. **(K)** LefSe analysis. **(L)** Heatmap analysis of the relative abundance of gut microbiota in the two groups at the family level. **(M)** Monofactor network analysis. **(N)** Dominance index analysis. Each boxplot represents the median, interquartile range, minimum and maximum values (n = 6). Compared with the control group, *P < 0.05, **P < 0.01.

The relative abundance of dominant microbiota is closely related to the changes in intestinal microecological function. Two groups of gut microbiota species with the highest relative abundance at the phylum and family levels were selected to determine the key microbiota affecting the onset of PD. Community bar charts can be used to compare the dominant species of gut microbiota and their relative abundances in different samples. At the phylum level, dominant taxa in both groups included Bacteroidota, Firmicutes, Actinobacteria, Verrucomicrobia, and Proteobacteria. Notably, Bacteroidota decreased (control: 58.65%, model: 43.76%), while Firmicutes increased (control: 32.87%, model: 39.20%) in the model group (**Figure 6A**). At the family level, the dominant intestinal bacteria with a difference of less than 0.05 between the two groups were mainly *Lachnospiraceae* (control group: 20.68%, model group: 5.67%) and *Erysipelotrichaceae* (control group: 5.04%, model group: 12.25%), *Akkermansiaceae* (control group: 3.42%, model group: 8.00%), *Bacteroidaceae* (control group: 0.22%, model group: 0.02%) (**Figures 6B, C**). Box-plot analysis confirmed significant differences at phylum and family levels. At the phylum level, compared with the control group, the relative abundance of Bacteroidota in the model group decreased (*P* < 0.01), while the relative abundance of Actinobacteria (*P* < 0.05) and Tenericutes (*P* < 0.05) increased (**Figures 6D–F**). At the family level, compared with the control group, the relative abundances of *Lachnospiraceae* (*P* < 0.01) and *Bacteroidaceae* (*P* < 0.05) in the model group were decreased, and the relative abundance of *Erysipelotrichaceae* (*P* < 0.05) and *Akkermansiaceae* (*P* < 0.05) increased (**Figures 6G–J**).

To further identify core taxonomic groups that may underlie intergroup differences between rotenone−induced PD mice and controls (**Figure 6L**), we performed linear discriminant analysis effect size (LEfSe) with an LDA score threshold of 4. This analysis revealed nine significantly different taxa at levels ranging from phylum to species (LDA score > 4, *p* < 0.05, **Figure 6K**). We also assessed sample-specific bacterial prevalence by calculating the coefficient of variation of dominance indices at the family level. This allowed identification of taxa dominant in some samples and rare in others. Notably, six families, including *Turicibacteraceae* and *Desulfovibrionaceae*, exhibited significant variation (**Figure 6N**). Among these, *Lachnospiraceae* showed a significant difference in relative abundance between groups (*P* < 0.01, **Figure 6G**). It is worth our further exploration of its classification significance.

Monofactor network analysis was used to analyze the correlations among microbiota. We constructed a family-level monofactor network using the top 30 most abundant species, selecting pairs with an absolute Spearman correlation coefficient > 0.5 (*P*< 0.05). Prior differential abundance analysis had shown reductions in *Lachnospiraceae* and *Bacteroidaceae* in model mice (**Figures 6G, J**). In the monofactor network, we found that *Bacteroidaceae* was positively correlated with *Rikenellaceae*, while *Lachnospiraceae* negatively correlated with *Erysipelotrichaceae* (**Figure 6M**). These associations further support the interrelated roles of these families in PD pathogenesis and suggest that they should be analyzed in conjunction.

### 3.7 Correlation analysis between gut microbiota and inflammatory indicators in rotenone induced PD mouse model

**Figure 7:**
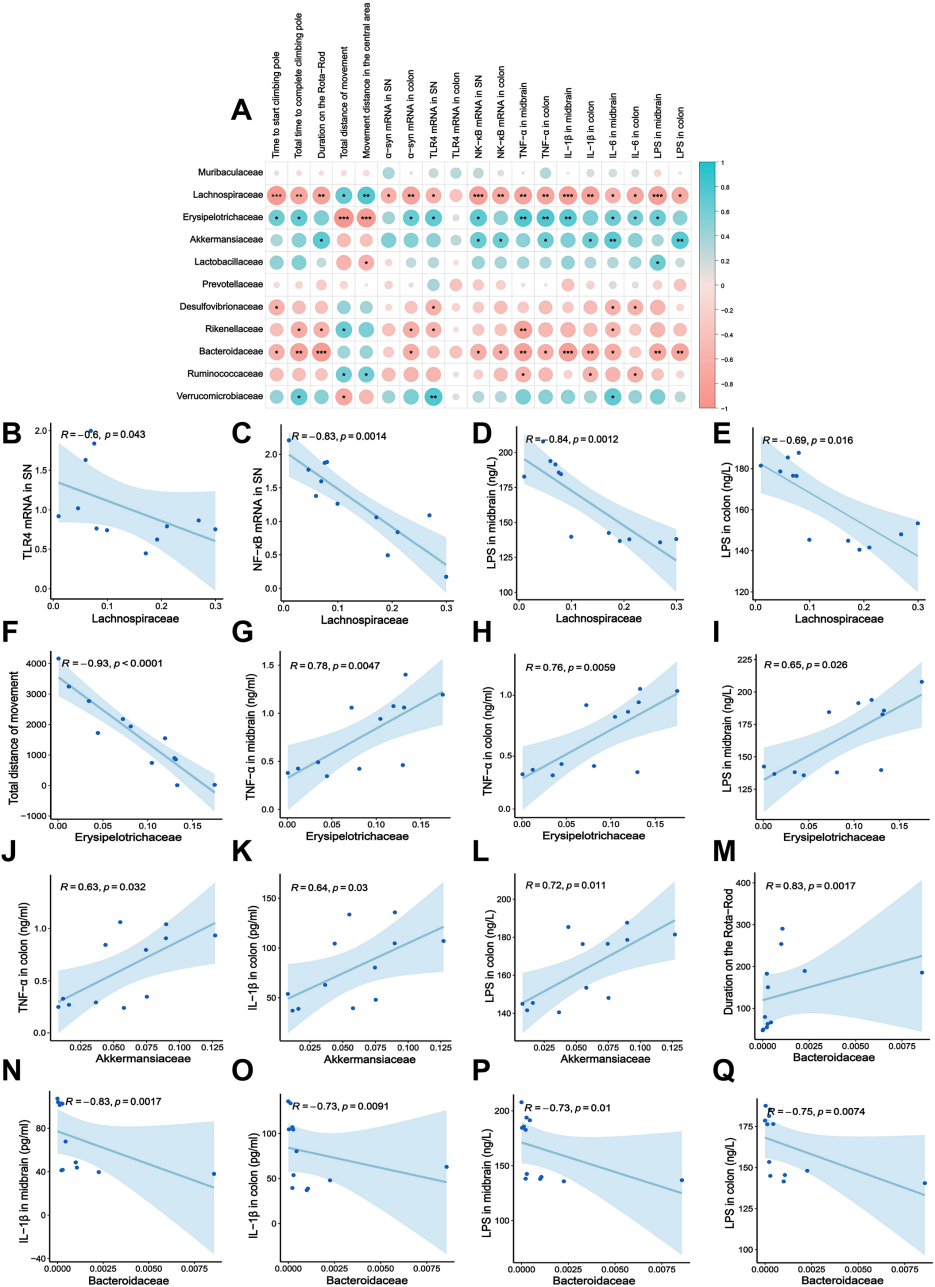
Correlation analysis between gut microbiota and inflammatory markers in rotenone-induced PD mouse model. **(A)** Heatmap analysis between major gut microbiota and other experimental indicators. **(B–Q)** The rest is the specific correlation scatter plot analysis between these several types of gut microbiota and other experimental indicators. *P < 0.05, **P < 0.01, ***P < 0.001.

In the rotenone induced PD mouse models, interactions between the gut microbiota and inflammatory indicators are mediated via the microbiota-gut-brain axis and collectively influence the onset and progression of PD. To further clarify the exact correlation between the 16S rRNA sequencing results and several other experimental parameters, a correlation heatmap diagram was selected for intuitive display. The results indicated that relative abundance of some bacterial families (such as *Lachnospiraceae*, *Erysipelotrichaceae*, *Akkermansiaceae* and *Bacteroidaceae*) was significantly correlated with behavioral outcomes (duration on the Rota-Rod, time to start pole climbing, total time to complete pole climbing, total movement distance, movement distance in the central area), α-syn mRNA expression in the SN of the midbrain, TLR4/NF-κB inflammatory pathway, and inflammatory indicators (TNF-α, IL-1β, IL-6, LPS) (*P* < 0.05 or *P* < 0.01 or *P* < 0.001, **Figure 7A**).

We then selected these four bacteria for further correlation analysis with specific inflammatory and behavioral indicators. The relative abundance of *Lachnospiraceae* was significantly negatively correlated with TLR4 mRNA (*R* = −0.6, *P* = 0.043) and NF-κB mRNA (*R* = −0.83, *P* = 0.0014) in the SN of the midbrain (**Figures 7B, C**). Additionally, *Lachnospiraceae* abundance was negatively correlated with LPS levels in both the midbrain and colon (midbrain: *R* = −0.84, *P* = 0.0012, **Figure 7D**; colon: *R* = −0.69, *P* = 0.016, **Figure 7E**). As demonstrated earlier, *Erysipelotrichaceae* exhibited a negative correlation with *Lachnospiraceae*. As shown in **Figures 7A, F-I**, the relative abundance of *Erysipelotrichaceae* was positively correlated with the TNF-α in both the midbrain (*R* = 0.78, *P* = 0.0047) and colon (*R* = 0.76, *P* = 0.0059), and negatively correlated with the normal motor function index (total movement distance) of PD (*R* = −0.93, *P* < 0.0001). The relative abundance of *Akkermansiaceae*, which showed significantly increased abundance in the model group compared to controls, was primarily associated with intestinal inflammation. Specifically, it exhibited positive correlations with TNF-α (*R* = 0.63, *P* = 0.032), IL-1β (*R* = 0.64*, P* = 0.03), and LPS (*R* = 0.72, *P* = 0.011) in the colon (**Figures 7J–L**). In addition, *Bacteroidaceae* abundance, which was significantly reduced in the model group compared to controls, was positively correlated with motor function index (duration on the Rota-Rod; *R* = 0.83, *P* = 0.0017, **Figure 7M**). Meanwhile, it showed negative correlations with IL-1β (*R* = −0.83, *p* = 0.0017) and LPS (*R* = −0.73, *P* = 0.01) in the midbrain, as well as IL-1β (*R* = −0.73, *P* = 0.0091) and LPS (*R* = −0.75, *P* = 0.0074) in the colon (**Figures 7N–Q**).

## 4 Discussion

In this study, a PD mouse model with both peripheral gut lesions and central neurodegeneration was successfully established by chronic neck and back injection of rotenone. The behavioral evaluation showed that the mice in the model group exhibited a shorter latency on the Rota-Rod test, longer latency and completion time in the pole test, and decreased total movement distance, central area movement distance, and entries into the central area. These results confirm that chronic rotenone administration induces deficits in balance, coordination, and spontaneous locomotion. These manifestations are highly comparable to the typical bradykinesia, postural balance disorder and freezing of gait in PD patients, suggesting that the model effectively mimics core PD phenotypes (22). Furthermore, histological analysis revealed pathological features of the model, with abnormal aggregation and increased mRNA expression of α-syn in both SN of midbrain and colon tissues. This finding is consistent with previous studies (23), which have reported that α-syn aggregation in the intestinal mucosa precedes motor symptoms in both animal models and PD patients (24). It has been proposed that misfolded α-syn may propagate to the brain via the vagus nerve, leading to dopaminergic neuronal degeneration, with gut microbiota dysbiosis being a potential trigger for α-syn misfolding (25).

Gut microbiota homeostasis and biodiversity critically regulate PD pathogenesis (26). Studies have shown that dysbiosis of the gut microbiota can release pathogen-associated molecular patterns (PAMPs) such as LPS. These molecules trigger myeloid differentiation factor 88 (MyD88) -dependent signal transduction by binding to TLR4 on the surface of enteric glial cells (EGCs) and lamina propria immune cells. PAMPs initiate promotes nuclear translocation of NF-κB, thereby upregulating the transcriptional levels of pro-inflammatory indicators such as TNF-α, IL-1β, and IL-6, and inducing chronic low-grade intestinal inflammatory response (10, 27). Subtained TLR4/NF-κB pathway activation inhibits the expression of intestinal epithelial tight junction proteins (such as occludin and zonula occluden-1), disrupt the intestinal epithelial barrier function, and lead to systemic translocation of LPS, inflammatory indicators and misfolded α-syn via the portal circulation. These circulating mediators induce matrix metalloproteinases (MMPs) expression within blood-brain barrier (BBB) endothelial cells, degrading vascular endothelial tight junction proteins, disrupting BBB structural integrity, and propagating neuroinflammation into the central nervous system, particularly targeting the midbrain SN (28, 29). Peripheral LPS and α-syn synergistically activate the TLR4/NF-κB signaling axis of microglia, driving microglial polarization towards the pro-inflammatory M1 phenotype, and releasing inflammatory indicators such as TNF-α and IL-1β, directly damaging dopaminergic neurons in the midbrain SN pars compacta (8, 9). Notably, α-syn can also descend to the intestine via the vagus nerve, further disrupting the intestinal microecological balance and forming a vicious cycle of peripheral-central inflammation (30, 31). This evidence highlights the critical regulatory role of the microbiota-gut-brain axis in PD. To further elucidate the inflammatory mechanism of gut-brain axis communication in rotenone-induced PD model, we focused on the activation status of the key TLR4/NF-κB signaling pathway and its downstream inflammatory responses. As confirmed by our molecular assays (RT-qPCR, western blot and ELISA), rotenone exposure significantly activated the TLR4/NF-κB pathway in both the SN of the midbrain and colon tissues in the PD mouse model, and increased the levels of LPS, TNF-α, IL-1β and IL-6.

Microbiota analysis indicates that the gut microbiota dysbiosis in rotenone-induced PD models may participate in the disease process by reducing diversity and altering the composition and structure of key microbiota. The alpha diversity analysis (Chao, Shannon, and Simpson indices) consistently showed significantly decreased richness and evenness in the model group compared to the control group, indicating that rotenone exposure led to impaired ecological complexity of the intestinal microbiota. The beta diversity analysis further confirmed the reshaping of the microbiota structure. These findings are consistent with previous reports demonstrating similar alterations in microbial communities in rotenone-induced PD models (10, 23, 32). At the taxonomic level, the PD model mainly presents an imbalance pattern of significantly reduced abundance of the Bacteroidota phylum level and increased abundance of the Actinobacteria phylum level. Clinical studies have similarly reported a decreased relative abundance of Bacteroidota in PD patients (33, 34). Bacteroidota and Firmicutes represent major constituents of the gut microbiota and maintain a symbiotic relationship with the host. Bacteroidota can promote the absorption or storage of energy in the body and participate in important metabolic activities in the colon (35). Some *Bacteroides* genus belonging to Bacteroidota can also produce short-chain fatty acids (SCFAs), increase propionate content, and play an important role in the integrity of the intestinal mechanical barrier and immune regulation (36–39). Conversely, Actinobacteria can serve as a potential taxonomic bacterial biomarker between PD patients and healthy controls. A metagenomic analysis study revealed that the relative abundance of the Actinobacteria phylum increased in PD patients compared to the healthy control group, with five candidate genera within this phylum positively correlated with clinical inflammatory markers of PD. These results indicate that the alterations of Actinobacteria may play a significant role in the pathogenesis and inflammatory response mechanism of PD (40).

The analysis at the family level revealed more pathologically significant changes. *Lachnospiraceae* and *Bacteroidaceae*, as major producers of SCFAs like butyrate and propionate, provide crucial anti-inflammatory signals. Their reduction diminishes this SCFA-mediated suppression of NF-κB activation and compromises gut barrier integrity. In the rotenone-induced PD mouse models, the relative abundance of the anti-inflammatory bacteria *Lachnospiraceae* decreased. Our findings are consistent with those of other studies, which also reported a decline in *Lachnospiraceae* and related genera in fecal samples of PD patients (41, 42). It has been suggested that reduced abundance of *Lachnospiraceae* may exacerbate intestinal inflammation, compromise the intestinal barrier, and promote the production of toxic metabolites, thereby aggravating and sustaining the clinical manifestations of PD (43, 44). Our correlation analyses further demonstrated a negative association between *Lachnospiraceae* abundance and inflammatory indicators. The relative abundance of *Lachnospiraceae* was significantly negatively correlated with the expression of α-syn and TLR4/NF-κB inflammatory pathways in the SN of the midbrain and colon, and was also negatively correlated with the levels of LPS and inflammatory factors (TNF-α, IL-1β and IL-6). Additionally, among the 9 differential markers identified in the LefSe analysis, *Lachnospiraceae* was included, underscoring its taxonomic significance in the PD model. The family *Bacteroidaceae*, belonging to the phylum Bacteroidota, exhibits a positive correlation with Rota-Rod test performance, suggesting its potential as a biomarker for motor function and recovery in PD. Furthermore, *Bacteroidaceae* exhibits negative correlations with levels of NF-κB mRNA, LPS, TNF-α, IL-1β in midbrain and colonic tissues, as well as IL-6 levels in the midbrain. *Bacteroidaceae* is predominantly beneficial to the host, possessing carbohydrate-degrading capabilities that enhance intestinal immunity and metabolic function through propionate production in the gut. These functions have been corroborated in studies focusing on the phylum Bacteroidota.

In our previous microbiota monofactor network analysis, *Lachnospiraceae* exhibited a negative correlation with the proinflammatory family *Erysipelotrichaceae*. Consistent with other reports, the relative abundance of *Erysipelotrichaceae* in PD mouse models was also significantly increased compared to healthy controls (45). This bacterial family is associated with systemic inflammation (46, 47), and in our analysis, its abundance was positively correlated with inflammatory cytokines and negatively correlated with motor function indicators. Another potential pro-inflammatory bacteria, *Akkermansiaceae*, also showed a trend of relative abundance increase in rotenone induced PD mouse models. This observation aligns with prior studies (10, 42, 45, 48). *Akkermansiaceae* and its genus *Akkermansia* may contribute to PD pathogenesis by degrading the intestinal mucus layer, compromising epithelial integrity, and facilitating the aberrant aggregation of α-syn in the enteric nervous system, thereby exacerbating disease progression (48, 49), which is highly consistent with the positive correlation between *Akkermansiaceae* and intestinal inflammatory indicators that we observed in the correlation Heatmap and scatter plot. The positive correlation between *Bacteroidaceae* and *Rikenellaceae* suggests a Potentially protective effect of *Rikenellaceae* on PD. Although previous studies reported decreased *Rikenellaceae* abundance in PD models (50), our data showed a non-significant reduction in rotenone-induced PD mice. Notably, *Rikenellaceae* holds unique taxonomic significance in PD gut microbiome studies (51), and was significantly increased following fecal microbiota transplantation (FMT) treatment (52). Contradictorily, other reports suggest an elevated abundance of *Rikenellaceae* in PD model group (53), and correlates with cognitive impairment in PD patients (54). These conflicting findings underscore the need for further investigation to clarify the precise role of *Rikenellaceae* in PD pathogenesis and therapeutic responses.

In summary, within the rotenone-induced PD model, we propose that gut microbiota dysbiosis, characterized by reduced relative abundance of *Lachnospiraceae* and *Bacteroidaceae* alongside increased *Erysipelotrichaceae* and *Akkermansiaceae*, disrupt the intestinal barrier integrity, and facilitate LPS translocation. This systemic translocation subsequently activates the TLR4/NF-κB signaling axis in both intestinal and midbrain SN regions, triggering enteric-brain inflammatory responses. Notably, microbial metabolites such as SCFAs and mucin degradation products, which serve as key mediators in the involvement of gut microbiota in PD pathogenesis, may further contribute to dopaminergic neuronal damage by modulating the TLR4/NF-κB signaling pathway. Emerging evidence underscores the intricate interplay between gut microbiota and immune regulatory mechanisms across multiple diseases, with the gastrointestinal tract serving as the primary interface for microbial-host immune interactions given its status as the body’s largest immune organ (5). Our findings emphasize correlations between these microbial alterations and inflammatory markers associated with TLR4/NF-κB pathway activation, prioritizing analysis of key microbial taxa influencing PD pathogenesis and disease outcomes. Beyond inflammatory mechanisms, critical processes such as cell death (particularly mitophagy, ferroptosis, and cuproptosis), mitochondrial dysfunction, autophagy, and oxidative stress provide novel research perspectives for elucidating the pathological mechanisms of PD.

Current PD management remains predominantly dependent on levodopa-based therapies. While providing temporary symptomatic relief, they fail to modify disease progression. Moreover, long-term use may aggravate the disease progression via pulsatile stimulation mechanism and induce serious complications such as dyskinesia and symptom fluctuations (55). Consequently, developing mechanism-based therapeutic strategies holds substantial clinical value. Microbiota-targeted therapies have emerged as promising research frontiers for PD. FMT, for instance, remodels α-syn-metabolizing microbial communities, restores gut microbial homeostasis, enhances intestinal function, and confers neuroprotection (10, 56). In addition, FMT can also alleviate chronic intestinal inflammatory responses by regulating the intestinal immune microenvironment, reducing the abundance of pro-inflammatory microbiota while increasing the proportion of anti-inflammatory microbiota (57). Beyond FMT, microbiota-modulating approaches, such as probiotic preparations (58), specific dietary intervention (59), regular exercise (60), and supplementary and complementary therapies like acupuncture (61), all demonstrate therapeutic efficacy via microbiota-gut-brain axis regulation.

Based on the intricate interplay between gut microbiota dysbiosis and multiple PD pathogenic pathways, our findings point toward highly specific bacterial taxa and their associated metabolic outputs as the most promising therapeutic targets. The restoration of SCFA-producing families, particularly *Lachnospiraceae* and *Bacteroidaceae*, emerges as a primary strategic goal, given their crucial role in maintaining gut barrier integrity and suppressing TLR4/NF-κB-driven neuroinflammation. Concurrently, interventions aimed at modulating mucin-degrading *Akkermansiaceae* to prevent barrier disruption and the systemic translocation of inflammatory mediators are equally critical. Therefore, these findings provide experimental rationale for therapeutic interventions targeting dysbiotic microbiota through FMT, probiotics, selective dietary modifications, or traditional Chinese medicine approaches. Future therapies should prioritize precision strategies. By modulating gut microbial composition to restore healthy relative abundances and maintain microbial equilibrium, such interventions may regulate immune function, improve systemic health, and offer effective therapeutic strategies for PD management.

However, several limitations of this study should be acknowledged. First, due to experimental constraints, interventional treatments targeting key microbial taxa were not investigated, leaving the therapeutic efficacy of such modulation undetermined. Additionally, the definitive causal relationship between microbial dysbiosis and TLR4/NF-κB pathway mediated inflammation requires validation through intervention experiments such as FMT or antibiotic depletion protocols. Second, the study was constrained by a relatively small sample size and limited scope of immune parameter assessment, necessitating expanded experimental investigations to comprehensively characterize microbial taxa and immune mediators implicated in PD pathogenesis. Additionally, a more comprehensive assessment of intestinal barrier integrity, including the expression of tight junction proteins such as occludin and zonula occluden-1, as well as improved quantification of neuronal damage markers such as helper T cell-positive immunohistochemical counts and levels of dopamine and its metabolites, remains necessary to enhance the reliability of experimental outcomes. Furthermore, given the complex compositional dynamics of gut microbiota involved in PD, the pathophysiological impact of individual microbial taxa appears limited, with disease progression likely driven by synergistic effects of multi-microbial interactions and inflammatory responses. This multifactorial interplay represents a critical direction for future mechanistic exploration and validation.

## Data Availability

The datasets presented in this study can be found in online repositories. The names of the repository/repositories and accession number(s) can be found in the article/**Supplementary Material**.
