# Supplementary material for: TLR4/NF-κB signaling-mediated neuroinflammation is associated with gut microbiota dysbiosis in a mouse model of Parkinson’s disease
Source: Front Immunol. 2026 Jan 30;17:1672241. doi: 10.3389/fimmu.2026.1672241 (PMC12901343; doi:10.3389/fimmu.2026.1672241)
Supplement: Supplementary file 1 [file DataSheet1.pdf]

Supplementary Table 1. Relative abundance of gut microbiota at phylum and family levels

Taxonomic level	Relative abundance (%)	Control	Model	*P*-value
	Bacteroidetes	58.65	43.76	0.006
	Firmicutes	32.87	39.20	0.150
	Actinobacteria	2.85	9.44	0.010
	Verrucomicrobia	1.88	4.12	0.406
**Phylum**	Proteobacteria	3.04	0.79	0.200
	TM7	0.48	1.73	0.150
	Tenericutes	0.036	0.54	0.025
	Cyanobacteria	0.010	0.018	0.222
	Deferribacteres	0.00099	0.001	0.674
	Muribaculaceae	47.73	51.63	0.307
	Lachnospiraceae	20.68	5.67	0.001
	Erysipelotrichaceae	5.04	12.25	0.013
**Family**	Akkermansiaceae	3.42	8.00	0.019
	Lactobacillaceae	1.57	3.11	0.262
	Prevotellaceae	1.59	1.36	0.707
	Desulfovibrionaceae	1.11	0.35	0.109
	Rikenellaceae	1.73	0.11	0.078
	Bacteroidaceae	0.22	0.02	0.016
	Ruminococcaceae	1.28	0.50	0.150
	Verrucomicrobiaceae	1.14	3.62	0.068

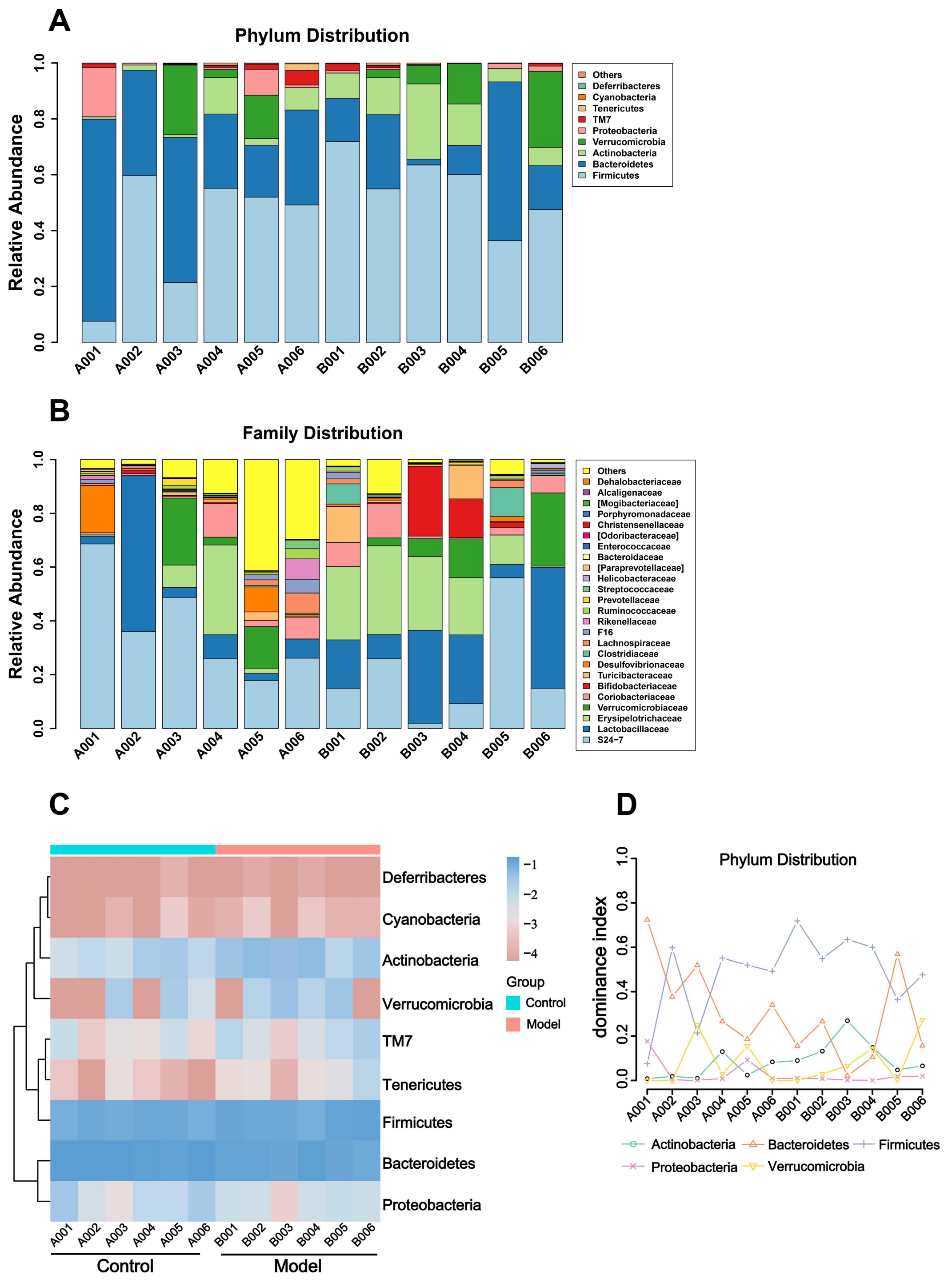

Supplementary Figure 1. Gut microbiota disorder in rotenone induced PD mouse model. **(A, B)** Bar charts of species composition at the phylum and family levels of each sample. **(C)** Heatmap analysis of the relative abundance of gut microbiota in the two groups at the phylum level. **(D)** Dominance index analysis at the phylum level.
